# On a Peculiar Form of Mental Alienation, Caused by the Political and Social Disturbances in France, during 1848

**Published:** 1863-10

**Authors:** 

**Affiliations:** Principal Physician of the Arbois Hospital.


### Art. III.—ON A PECULIAR FORM OF MENTAL ALIENATION, CAUSED BY THE POLITICAL AND SOCIAL DISTURBANCES IN FRANCE, DURING FEBRUARY, 1848.

By Dr. Bergeret, Principal Physician of the Arbois Hospital.*

*(Translated from the French by* Dr. Footman.)

The experience of past ages shows that the disease known under the name of mental alienation changes character with the times, and reflects, like a faithful mirror, the ideas peculiar to each

---

* *Annales d'Hygiène Publique.* Juillet, 1863.

epoch in the life of a people. What, indeed, is madness, but the exaggeration of a prevailing idea—the loss of balance of the faculties produced by a tension of the brain, so powerful that the natural equilibrium of its functions is disturbed?

There is one circumstance which renders the study of mental alienation full of interest to the philosopher and the physician: the madman, penetrated with the same ideas, and animated by the same passions as the sane man, exhibits his moral nature in all its nakedness. With him *amour propre* and fear of the world's opinion are destroyed, social considerations are extinguished, the passions are no longer surrounded with the charm that renders them seductive, the deceitful veil which hides from our eyes their perfidy and wickedness is torn away; the madman, abandoned to his ruling passion, led on by an irresistible impulse, forgets the rest of the world, tramples under foot all rules of decorum, and lives in himself and for himself alone. We are thus enabled to penetrate into the depths of his mind, and probe its most secret recesses. Madness exposes all the vices and all the weaknesses of the human heart—true death of the reason, it exposes to every eye the secret passions which have destroyed or disturbed the most noble of our faculties; even as the opening of the body after death reveals the secret lesions which have drained in silence the springs of life.

Regarded from this point of view, madness becomes one of the most interesting and truthful pages of the history of the mind of man. There are times when the number of victims who become a prey to this frightful disorder is increased in a terrible manner, and when the disease presents peculiarities worthy of special study. Among such periods may be reckoned the time of the Revolution of 1849. A kind of febrile agitation was felt in all the members of the social body; ideas the most strange, theories the most fantastical, were disseminated among the populace by vain sectaries, who sought only to realize the criminal dreams of a boundless vanity and an insatiable ambition; and they gained an easy victory over the inexperienced masses, for the ignorant multitude seized with avidity upon the deceptive bait which flattered all their worst passions. Many weaker minds also gave way, overcome by the fears excited by the menacing voice of parties and the howls of faction. The cases of mental alienation were multiplied greatly; in my own experience I met with at least ten times the number that occur in ordinary times.

All the cases of madness presented a peculiar type—doubtless impressed upon them by contemporary events. A new form of insanity seemed to have appeared in the world, and Dr. Groddeck, a German physician, published at this time a pamphlet entitled

*De Morbo Democratico, nova insaniæ forma.* I propose to give an account of several cases of this peculiar kind of lunacy coming under my own observation. The history of each case will be drawn from nature.

CASE I.—Victorine U., thirty-one years of age, married, in easy circumstances, of quiet disposition, inclined to melancholy, uneducated, performing her domestic duties with regularity. Dark, the features regular, the eyes large and black. Her manner is absent, and she walks with her eyes fixed on the ground, but raises them at times to heaven, with an air of inspiration and mysticism. She lives in a street the scene of considerable excitement during the Revolution of February. Immediately in front of her house was a seat, frequently used as a place of meeting by the revolutionary leaders of the faubourg. Victorine paid marked attention to their exciting discourses, and her mind became by degrees much affected by them, though nothing very extraordinary was noticed in her conduct till the time of the Bourges trial, when one day she suddenly left her home. Her husband found, on inquiry, that she had taken the diligence to Paris. He immediately followed her, and, on application at the police-office, found that, on her arrival in Paris, she had declared herself sent by Jesus Christ to deliver the prisoners of Bourges, and claimed to be immediately conducted to them, that she might accomplish her divine mission. The police put her into a carriage, and conveyed her at once to the Salpêtrière, where she was placed in the wards allotted to the insane. She was given up to the care of her husband, and taken back to her home and family. From the time of her pilgrimage to Paris, her mental state deteriorated rapidly; she spoke of nothing but the accomplishment of her glorious destiny; she passed whole days at her devotions; her lips moved always as if in prayer; neither the caresses of her children, nor the cries of her young infant, deprived of its mother's breast, were sufficient to withdraw her attention from the subject of her melancholy pre-occupation. One morning, about a month after her return from Paris, she presented herself in my consulting-room. "I am come," said she, "to ask you to give an order for the gendarmes to take me to Paris." "What do you want to do in Paris?" I demanded. "I am going to set free my friends, my children, the political prisoners." "And why do you undertake that duty?" I inquired. "I am the mother of the country—the predestined mother. Christ has commanded me to rescue them—He appears to me every night—He urges me on—I have no time to lose; I implore you to send me at once to Paris." "But what will become of your children when you are away?" I said. "Is not your first duty to look after them?"

"My children," she replied, "I sacrifice for my country's good; all is lost if I do not do as I am commanded; I am the Mother of the Republic; the political prisoners are my true children, and I must break their bonds. Let me start at once, I beseech you; I have no money, but I am told you can send me at the expense of the republic." "Do you know the names of those you would rescue?" I asked. "Know them!" she exclaimed—"the first is Drolin (Ledru Rollin), then there are Barabès (Barbes), Balanquin (Blanquier), Cachepail (Raspail), and Petit Blanc (Louis Blanc)—with them I must save France. We will destroy Despotism, the *Reds* must crush the *Whites*, or the greatest misery will overtake the poor people. I only can prevent it; God revealed it to me at Paris during my martyrdom." Every now and then she broke off the conversation, and appeared to listen attentively to some mysterious communications, her expression at these times wore an appearance of terror and exaltation indescribable, and she murmured rapidly some inarticulate sounds; sometimes she seemed to pronounce some cabalistic phrases which gave her the air of a sorceress making her incantations. At such moments, when I asked her what she was doing, she replied, "I am saving the people." Our interview was brought to a sudden close, by her rising quickly and saying with the air of one inspired, "Hark! the trumpet for the last judgment! I must hasten to deliver the political prisoners, or all will be lost." She then strode rapidly away.

Case II.—Louise N., aged forty, a widow with two children; fair, features delicate, dark blue eyes full of vivacity, and a fine forehead. Her expression usually open and lively nervous; and very intelligent. She is a needle-woman, who has been much tried by domestic misfortunes, and is evidently in reduced circumstances.

Until the Revolution of February, she had always shown herself a good mother and an excellent workwoman, but about this time she began to read newspapers of the most violent political character—they were to be found everywhere in those days, even in the most humble workshop. Louise passed whole nights in studying and paraphrasing, after her fashion, the numbers of the paper called *La Reforme*. The excitement which took possession of her mind soon became evident by the manner in which she repeated in the streets, to whoever would listen, the passages of the journal which had made the most lively impression upon her. Her good memory enabled her to reproduce them with great fidelity. She delivered them with much fire, in a tone of declamation, and with a fecundity of comments that seemed inexhaustible.

In a very short time her political pre-occupations made her neglect completely the duties of her profession and her domestic cares. Her time was occupied in preaching, as she said, for the regeneration of the human race. She was seized sometimes with fits of fury which disconcerted the whole household. I was one day a witness of one of these terrible scenes, and I will try and sketch the principal features for the benefit of the reader.

I found her in a wretchedly-furnished room, seated on the bed in her night-dress, her hair dishevelled, her eyes staring, and her features horribly distorted. She was gesticulating wildly, storming with passion, and rousing the whole neighbourhood by her savage cries. Her relations and children were weeping around her bed, and doing their utmost to calm her. "God has gone mad," she cried; "I will take his place. I must do my duty—I am God—I have received full powers—the face of the earth must be changed—the great ones shall be destroyed, and the poor shall take their places. I see the world ravaged—blood shall flow like water. Let the rich bring their money; the time is come for it. There shall be weeping and gnashing of teeth. No more misery—no more slaving of man for man—no more rich—no more gendarmes. Man should govern himself. I will save the country—the world shall be regenerated. I hold the judgment-book of the Republic; *I hold the wheel which shall crush the world. I have seen the mountain from whence come the stones that shall grind the rich ones of the earth.*" Having pronounced these words, she threw herself on her knees upon the bed, and cried out in a tone of voice of which no description can convey any adequate idea, "Oh God, I pray you preserve me from riches, for all the rich shall be damned—all shall perish miserably. Oh, what misfortune to be rich! Oh, my children, may God keep riches far from you, or you will be lost! I prefer poverty—I love it! I love it!—poverty should be mistress of the world. *The tavern before the palace.* The Pope is the beast of the Apocalypse. Religion is only fanaticism—the priests care only for money. Down with religion! down with the Pope! down with the priests! Robespierre, Proudhon, Ledru-Rollin, they are the true priests of humanity." I interrupted her suddenly by saying, "And Saint Vincent de Paul! come, what do you say of him?" "Shame!" she replied—"he is a Jesuit—down with the Jesuits!" Her weeping children threw themselves into her arms, and sought in vain to appease her; she put them violently from her, calling out, "No more children! no more relations! I know you not; I cannot be calm; I obey an irresistible power! I see visions—I am the Liberating Mother—I see in the papers things which I alone can understand. My son, who is dead, should change the face of the earth. I now am charged with this mission. I am the Apostle —the Messiah of the Democratic and Social Republic. Obey my commands, or the world is lost for ever!"

It is impossible to convey an idea of the melancholy group presented to my view, as this woman stood like a Pythoness, raging and with dishevelled hair, surrounded by her disconsolate friends and despairing children, who were seeking in vain to quiet her, and to drown in their tears the fearful blasphemies which she poured forth with a volubility that seemed inexhaustible.

CASE III.—One evening in June, 1849, I was sent for in great haste to see Marguerite N., who was said to be suffering from apoplexy. She is a widow, aged forty-nine, dark, thin, and bilious, very nervous, with a lively imagination and a good memory, but of unsound judgment. She is one of a family of artisans, and has no children. Has always been very fond of reading, but has read indiscriminately, and her passion for books has proved injurious, by filling her mind with a quantity of crude and confused notions. She has always been very vain; and although possessing sufficient means to supply all her wants, regarded with envy those whose fortune was superior to her own. This jealousy betrayed itself by declamations against the great, the rich, and people of rank, while, at the same time, she cried up persons of her own and of inferior station in life. When the Revolution of February broke out, she received with joy the torrent of strange and subversive ideas with which the country was inundated; she read with zeal the most demagogue journals and pamphlets. The absurdities upheld by all the sects of socialists, communists, and Fourierists, were for her the most sacred dogmas.

And here let me be allowed a word upon the nature of these doctrines, that the reader may be better able to realize the mental condition of Marguerite. These social heresies, borrowed from the most sensual form of paganism, proclaim a morality quite opposed to that which is taught by the evangelists.

Christ, who knew the weakness of our nature, its imperfections, its vicious propensities, which are and ever will be the principal source of evil, of injustice, and of the inequalities with which all human society is stamped—Christ taught resignation in adversity; he raised to the rank of the most sublime virtues, forgetfulness of oneself, austerity, patience in suffering, and contempt for material enjoyments. He said that the life of man was but a voyage—a time of trial—and that *his kingdom was not of this world.* Nothing can be more elevated, more pure, more spiritual than this doctrine. The morality of modern sectaries, on the contrary, is borrowed from the most rude and stupid sensualism. Completely losing sight of the imperfections of our nature, they affect to be able to realize in this world the dream of *perfect happiness.* They endeavour to increase the desire of the people for material enjoyments, and dazzle them by presenting to them a state of society in which man shall have everything that he can desire, without the labour of acquiring anything; it being the duty of the state to provide for all. They have erased the old proverb, so full of truth, *Help yourself, and Heaven will help you.* They proclaim imperturbably *the abolition of poverty, the extinction of pain.* They will decree one day that all the world shall be happy, and that suffering is exiled from our planet. This abominable charlatanism, despite its transparency, has duped and ruined thousands. The subject of the present observation will furnish us with an example.

I immediately hastened to Marguerite, and found her in the arms of two men, who restrained her with much difficulty. She was the subject of the most violent hysterical fit that I have ever witnessed. Her limbs stiffened convulsively; her face was horribly distorted; her respiration was accompanied with a loud guttural râle, and she ground her teeth so frightfully that the women who surrounded her were petrified with fear. I made her inhale some ether, and in a little time she recovered the use of her speech. The first words she uttered were these:—"Fetch me here those brigands of gendarmes—let them come! I will break them like glass—I will tear out their bowels! To lead about men in that manner, with a chain around their necks!—ah! what horror! what ignominy! This is the way the *Whites* show their fraternity!—they shall be accursed!—the people shall crush them in their anger."

I took one of her relations aside, and asked for an explanation of the strange words I had just heard. He informed me the attack had been brought on by the sight of a coach passing in the street, containing three prisoners in chains, accompanied by some gendarmes. She had had attacks of the same nature, but less powerful, produced before by the same means. He told me, also, that she raised her head, and became very excited habitually at the sight of an elegantly-dressed lady, a servant in livery, or a brilliant equipage; and that even, at times, when no external object could inflame her imagination, as in the middle of the night, she could be heard talking aloud, declaiming and vociferating violently.

I returned to Marguerite. She was less convulsed, but she was evidently in a state of great exasperation. She was in the midst of a most animated monologue:—"What an abomination thus to humiliate man, to put a chain round the neck of the most beautiful creation of God! to load citizens, republicans, with irons! When will the people break open the doors of the prisons and the hulks? No more executioners! no more victims! Victor Hugo has said it. All this shall disappear; there shall be no more poor; all men should be equal. Socialism will render them all equally happy. Why should one workman earn more than another? It is shameful. All should be equal. No more rich! no more poor! equality for ever. The old world is finished, my friends. A new Messiah is come. Louis Blanc will ensure bread to every one for his children. He is my God, my idol. He appeared to me in the night, his face shining with light."

I left her in the midst of these ravings, as I was anxious to learn who the three prisoners were who had just passed through the town, whose fate had so affected the heart of Marguerite, and thrown her into a state of excitation, of which my description can convey but a faint idea. I proceeded at once to the house of detention, and asked the jailor the names of the new comers. On consulting the registry, this is what we discovered. One of them was a professional thief; the second, an incendiary, who, for revenge, had set fire to the house of one of his neighbours, and suffocated a child in the flames; the third was the father of a family, who had endeavoured to violate his own daughter. Such were the three estimable citizens, the sight of whom had had so powerful an effect upon the nerves of Marguerite. See how the abominable doctrines which tainted the atmosphere at this period perverted the most natural sentiments, even those of pity.

I could enumerate many more cases of madness in which the subjects of mental aberration were almost exclusively of a political nature. But the preceding facts will suffice to show to what extent the new notions, thrown imprudently among the masses, corrupted the natural ideas and sentiments of the people. Indeed, the examples which I have cited do not represent an exceptional or isolated mental disposition among the people. How many were there at that time haunted by similar ideas, without, however, being disturbed to such an extent as to come to an actual outbreak of insanity? The immoral maxims taught by the modern innovators circulated among the population like a pestilential miasm. Many hapless individuals seized on the fatal germs, and nourished them in silence. But the insane are the *enfants terrible* of the great human family. They divulge involuntarily the passions which undermine in more or less secrecy the minds of those who are sufficiently strong to prevent their outward explosion.

I must now describe another modification that insanity has appeared to me to undergo in consequence of the events which took place after the Revolution of February.

The nation was divided into two hostile parties, who fought many a bloody battle in the streets of our cities, and strove not less ardently, though in a less terrible manner, in the arena of parliament, with the pen of the journalist, or in the electoral urn. The threats of parties and the sinister predictions of political Jeremiahs were echoed even in our smallest villages. Many weak imaginations were affected by them, and they were the cause of a great number of mental alienations of which the leading feature was a gloomy pre-occupation. Sometimes the victims were a prey to the darkest melancholy; at others, the fear of imaginary dangers pursued them, and they were tormented by terrible hallucinations or dismal phantoms. Several cases of this kind came before me. I will relate a few of them.

CASE IV.—Jean Pierre N., carpenter, an excellent workman, with a good character, caring for nothing but his work; good-tempered; not in the habit of frequenting the public-house; never interested himself in politics, and always turned a deaf ear to all the insinuations of political demagogues; refused to subscribe to any of the newspapers which excited to riot, to join any of the secret societies. On these accounts he had often to endure the taunts and menaces of his socialist neighbours; and we shall see in the sequel what a fatal effect the latter had upon his mind.

One day, as I was passing before his house, he called me in, saying that he had wished for some time to speak to me about his disease. "What disease?" I said; "you have an appearance of health and strength which many might envy." "Nevertheless," he replied, in a melancholy tone, "I am seriously ill." "Well, tell me, then, what is the matter with you?" "Sir," said he, "my whole body stinks horribly." I began to laugh; I saw at once that it was the mind, not the body, which was affected; for he was a man of very strong constitution and robust physical health. "You don't believe it," he continued; "and yet every one says so." "You imagine it," I said. "I beg your pardon," he replied; "I hear it distinctly. If I appear in the street everybody cries out as I pass by, 'Ah! how he stinks!—oh! he is rotten!' All the day people come and cry under my windows, that I am polluting the country, and that I ought to be thrown into the river. Twenty times during the night I am awakened by the same cries, so that I never go to bed without my sword. I dare not go out any more. I have no longer the heart to work. Cure me, I pray you, for I am very wretched."

I tried all kinds of arguments to persuade him that he was the victim of a delusion; that the words which he thought he heard were quite imaginary; that he was in a kind of waking dream. Nothing could convince him; and just as I was going away from him he repeated, with an air of consternation, and in the attitude of one who is conscious of a most disagreeable sensation—"Hark! Do you not hear some one now crying out that I must be thrown into the sewers?"

Case V.—Françoise U., a peasant girl, thirty-five years of age; artless and ignorant; of dark but pale complexion; black eyes, and a decided expression of melancholy. She came to me one day at Arbois, and said—"Sir, I wish it were possible for you to cure me, but I am afraid to tell you my disease." "My dear child," said I, "I am no prophet, and cannot possibly cure you unless you tell me what is the matter with you." "But it is such a singular disease," she said; "I do not understand it myself. Oh! I shall never have the courage to confess it to you: and, nevertheless, if you knew how I am tormented, I am the most miserable person in the village." "Come," I said, "have confidence in me, perhaps I shall be able to cure you."

"Ah, well," she replied, "believe it if you will, but I can no longer endure the houses of my village should be so badly built. This idea haunts me incessantly, I cannot look at the houses without shaking with fear. I am certain that they will fall down upon me; and I pass all my time in the fields because I dare not go home to my house for fear of being crushed to pieces. This is all my disease; cure me, I pray you. I cannot endure it any longer." "But," I said, "where have you got these strange notions from? Have you been hearing any alarming stories; or reading an account of some earthquake, perhaps?" "Oh! I can hardly read at all," she said; "but stay; I will tell you what happened to me at the time that so many oaks were being planted in the village. I had been to market to sell my butter, and was returning quietly home, when a man slipped into my basket a large printed paper that I had the misfortune to carry home. Oh! how I wish I had thrown it into the river as I went along, for I am sure it must have come from hell. My brother, who knew how to read, took it and read it out aloud. Oh! sir, you cannot imagine what there was in that paper. It said that the world could not last much longer as it was, and that a new one must be made; that the middle class and the rich had had their day, and that they must be got rid of; that the priests deceived the people—and ours is such a good man, who deprives himself of every comfort for the sake of the poor!—and our master's wife, who is always to be found at the bedside of the sick, who is their guardian angel, and never comes to them empty-handed! In short, this horrible paper prophesied the greatest misfortunes; and since that time these wretched ideas have seized hold on me. I never sleep. I, who formerly worked like two people, am no longer able to support my poor mother, because I dare not remain in the house. I believe that nothing is well-made in the whole world, and our house is so badly built that I am sure it will fall down and crush me in its ruins!" Scarcely had she finished these words than she burst into tears, and her voice was for some time drowned by her sobs. I could not restrain my feelings at the aspect of this innocent victim of our revolutionary orgies. "Is it possible," I exclaimed, "that a corrupting and shameless press should throw trouble into the hearts of these simple people, whose pure and unsophisticated innocence ought to have sheltered them from its baneful influence?"

I endeavoured to comfort Françoise and make her understand into what a deplorable error she had fallen. She seemed at the time to have implicit faith in my remarks; her face brightened up somewhat, and she left me apparently determined to put away from her the terrible delusions which had taken possession of her mind. I met her several times afterwards, however, and she reproached me in a sorrowful tone. "Oh! sir, you promised to cure me, but my disorder has returned, and I am more wretched than ever. No; I shall never again be able to look at the houses of our village without a shudder."

Case VI.—Antoine N., a vine-dresser, forty-five years of age, thin, nervous, of robust constitution, and great physical strength; uneducated, but with small abilities. His conduct was always bad. He was a frequenter of public-houses, and his wife is said to have died of grief at his behaviour. He has several times committed acts which have brought him into the hands of justice. Formerly he had sufficient to live upon comfortably, but through extravagance he lost nearly all his property. Here, then, was a soil well prepared to receive the seeds of communism. After the first days of the Revolution of February, he became more unsettled and excited than ordinary, and might be seen drinking wine nearly all day long. He was eager to see the fulfilment of the pompous prophecies which he had heard proclaimed in the clubs and the public-houses. He eagerly desired that the time should come for the division of lands, and the printing of assignats. He had particularly grieved at the loss of a piece of land which he had been obliged to sell to satisfy some of his creditors, and he nourished a secret hope that somehow, when the division of lands took place, his former piece would be allotted to him—and then he had many a troublesome creditor that he would be glad to satisfy with those rags of paper

(assignats). In fact, he believed very sincerely for some months that his dreams would be realized, so seductive were they; but when he saw that after a time events took quite a different turn from that which he had expected, so great a revulsion took place in his feelings that he became ultimately quite insane. He thought the principles of communism had not advanced because the "*Whites*" had been able, by means of witchcraft, to prevent their success by sending over the country a legion of evil spirits. "I have," he said to me, "constantly an evil spirit in each ear, crying out that I am a worthless fellow, a wretch, a Jacobin, a drinker of blood, and all kinds of nonsense. They do not leave me a minute's repose. I have hardly got to sleep at night before I am roused up by their cries. But it is not that which torments me most of all. They swarm all around me; they play me all kinds of tricks, and persecute me incessantly. If I go to the cellar to draw some wine, they upset it; if I light a lamp, one of them comes behind me and blows it out immediately; scarcely a day passes without their putting something nasty into my soup. When I eat, they make me drop the piece that I would put into my mouth, and will not let me have a meal in quiet. Indeed I should never finish, were I to relate all the trouble they cause me." "And who has let them loose against you in this manner?" I demanded. "The *Whites*—the *Whites.* They know that I detest them. They saw that we were going to be masters, and so they have made a bargain with the devil, who has sent all these evil spirits. Here," he said, with a frightened air, showing me one of his ears, "here is one who has nestled on this side, and who is calling out, loud enough to deafen me, all kinds of impudent things." My efforts to disabuse him of his delusions were unsuccessful. From the time I first saw him he became worse and worse. The hallucinations as to the presence of evil spirits in his ears were so powerful, that he tried all kinds of means to stop up his ears: he plugged them with earth, with cinders, with plaster; once he even tried to build them in entirely with mortar, another time to cut them off with a razor; but nothing seemed to lessen these painful impressions. He was often heard going about the house all night long in pursuit of the evil spirits. The voices sometimes seemed to become so unbearable to him, that he had been known to lie for twenty-four hours in bed, with his fingers in his ears, and refuse food and drink of any kind. He was ultimately taken to the asylum of Dôle of which he is still an inmate.

Case VII.—Thérèse N., aged twenty-three, amiable, diffident, a good mother, and an excellent wife; her husband a vinedresser, honest and industrious, who took no active part in political matters. She had the misfortune to live in a quarter where political discussions were frequent, and the sounds of quarrelling and angry threats against her husband often reached her ears. She was very weak and timid, and this state of affairs produced a most painful impression upon her.

Passing one day through the street in which she lived, I saw Thérèse sitting in front of her house with her little one in her arms, and I was struck with the profound melancholy of her aspect. I went up to her, and said, "What ails you, Thérèse; you seem to me to be very unhappy?" Instead of answering me, she burst into tears. I waited a short time, tried to calm her, and urged her to tell me the cause of her sorrow. "Oh!" she exclaimed, at last, "it is very sad, but I will tell you all, and perhaps you may be able to ease me; but promise me to say nothing of it to my husband." "I promise you." "Can you imagine, that I hear voices which cry out constantly to me, 'You are ruined, your husband will be killed, and you with him. Take care of yourself, take care of your child, take care of your husband.' Night and day, in the country, in the church, these voices pursue me, and do not leave me a moment's peace. I have no sleep, I have no heart to do anything, my milk is spoiled, and my poor child dwindling away—and yet I dare not tell my husband, for fear of troubling him. He sees that I am suffering, but does not know what is the matter with me, and is vexed at not being able to comfort me. Oh, cure me, cure me, I beseech you, if you can!" At this moment a little dog came out of the house, and began to bark. An expression of terror came over Thérèse which immediately attracted my attention. "What!" said I, "you are not afraid of this little dog?" She looked confused, blushed deeply. "Well, I will confess it," she said, "though, I know, it will seem very absurd to you; and yet I cannot help it, but every time this dog barks it seems to me to cry, 'You are about to die.'" At these words, she recommenced weeping, and bathed the face of her little one with tears. I myself was so overcome with emotion, that I was scarcely able to give her a word of consolation, but went away promising to come again shortly to do the best I could to cure her. A few days after I saw her again, she was rather more composed, and she had drowned the dog.

Case VIII.—Gabrielle N., wife of a vine-dresser, fifty-two years of age, of nervous temperament, irritable, melancholy, and bad-tempered. After the Revolution of February, her house was the favourite place of resort of the women of the neighbourhood; they held there a kind of female club. It was doubtless on account of the extreme nature of her political opinions that her house was chosen as a place of meetings. She used to read aloud the most violent passages of the revolutionary journals, and comment upon them in language so furious as to be worthy of one of the famous *Knitters* of '93.

In 1850, long after peace was restored, and the club dissolved, she preserved all her former vehemence and violent political aspirations : but it was impossible for her nervous system to resist for an indefinite space of time such a state of continual and marked excitement. She was seized one night with a violent fit of hysteria, which left her for some days after in a state of complete and melancholy prostration. From that time her ideas took another direction—the fear of death seized on her, and she fell into a state of melancholy and hypochondriasis. She believed every moment she was about to die, bade farewell to her friends, and made arrangements for her burial. I prescribed some medicine for her, but she refused to take it, saying it was useless. She repeated incessantly that she was lost. Whenever the bell of the parish church sounded, she said it was her funeral knell. She heard voices crying in the street that her end was near, that her body was decomposing, and that her carcase was only fit to be thrown to the wolves. These hallucinations became so troublesome that she was obliged to remove from her usual bedroom, which looked upon the street, to a small garret in the back of the house. At the end of some months her mental alienation took another form. She fancied that one of her daughters who had taken much care of her during her illness, and of whom she was very fond, had been threatened with arrest, and was in danger of being taken to prison. She was so much under the influence of this prevailing idea, that she was always crying out, "They want to take my daughter, but I forbid it; they shall not take her, poor girl, what wrong has she done?". Every moment she hastened to the window to see if the gendarmes had come to take away her child. It was impossible to disabuse her of the painful notion; and this childish fear, which had so taken possession of her, led her by a fatal induction to meditate a frightful crime. After remaining in the most melancholy state for nearly six months, her countenance one day brightened up suddenly with a smile of joy, as if some ray of light had traversed her soul, and she cried out, "Thank God ! I have just found out how to prevent their taking away my child. They shall not have her ! they shall not have her ! I will kill her ! yes, kill her !" and her face beamed with a look of horrid satisfaction. Her friends knowing the state of mind she was in, did not attach much importance to her statement, but a few days afterwards, the inhabitants of the opposite house were roused up in the night by the appearance of a lurid flame at the windows of the room of Gabrielle and her daughter. They hastened to the house, and proceeded at once to the room, and on entering it a most frightful spectacle was presented to their view. The bed of the daughter was on fire, the poor child had just woke up, and was half-suffocated by the flame and smoke; and her father, aroused by her cries, was making every possible exertion to revive her. Gabrielle, in her chemise, stood still in the middle of the room, repeating, with a fiendish laugh, "Ah! ah! well done. I have just suffocated her. They may come for her now, but they wont have her."

In fact, seizing the opportunity when her husband and the other children were fast asleep, she had risen quietly, piled a heap of straw under the bed of her sleeping child, and set fire to it. The neighbours hastened to extinguish the fire, and the day after showed me the charred remnants in a corner of the garden. She is at present in a state of prostration, bordering on dementia. Nothing will arouse her from her torpor; her faculties are blunted, and she leads a vegetative existence, hardly differing from that of brute beasts.

Case IX.—Joseph N., forty-eight years old, strong constitution, bilio-nervous temperament; his temper very hasty, and excitable; married at thirty-two years of age; his wife a very superior woman; one child only the result of their union. In starting in life he had only just sufficient capital to furnish a small hosiery shop. By dint of industry and perseverance in the course of a few years his business increased, and he was able to purchase some property with his savings which, year by year, became more considerable. When the Revolution of 1848 broke out, the following was his financial position. He possessed, first, several good pieces of land; secondly, the house which he inhabited; thirdly, a large number of small sums due to him from his creditors; and fourthly, a purse containing 1600 francs, which he kept most carefully locked up, and which plays a prominent part in the history of his disease. His debts were quite insignificant.

One would have thought that, in the prosperous condition in which he found himself, his own instinct would have taught him to join the side of order and conservatism. The line of conduct which he followed, however, was just the reverse of this. Three principal circumstances require to be dwelt on to explain his conduct on this occasion. First, he was naturally very vain; and since the increase of his property his pride had also very much increased. His whole bearing was indicative of the satisfaction with which he contemplated his rising fortune; what he had already acquired served but to augment his appetite for more; and he had been often heard to remark, on reading the journals which declaimed against great fortunes, that if a reign of terror like that of '93 should compel the great proprietors to emigrate, should cause the sequestration of their goods, and give rise to the creation of assignats, he should be able with his bag of money to procure large quantities of these bits of paper, to buy, at a very low price, the goods of the emigrants, and very quickly to become a rich man. Influenced by these ideas, Joseph, the small proprietor, threw himself body and soul into the socialist crusade. He became one of the most virulent detractors of the large proprietors of his neighbourhood; he excited the people constantly against them, declaring that the time had arrived when everybody should be rich, and that riches should no longer be confined to a privileged few. I ought to have explained that, before his marriage, Joseph had served for five years as coachman in a very rich family. This residence seemed to have left some indelible impressions upon his mind. First, the luxury that reigned there seemed to have laid the foundation of his ambition; and secondly, the haughty bearing of the mistress of the house left in his mind a feeling of rancour and bitterness against the great, which did not by any means subside with time.

Envy and hatred were the two passions which animated him in his implacable war against those whose property he coveted; and to these may be added a third sentiment—namely, the secret hope, that by ranging himself on the side of the communists, when the great crisis came, his friends and brothers, in the midst of the general havoc, would spare his possessions. Finally, one other circumstance will serve to complete our explanation of his conduct. He read only the socialist journals, and as he was too ignorant to distinguish truth from falsehood he gave the most implicit belief to everything which appeared in their columns. From the date of the Revolution to the end of 1849 he continued in this state of mind; but seeing that the course of events did not answer to his expectations, and that the chances of a fresh revolution diminished daily, his mind overflowed with bitter vexation. He became melancholy, dreamy, distracted, and taciturn; he hardly ever alluded to politics, and if, in spite of himself, he was led on to this subject, he expressed himself with so much anger, that it was evident his reason was even then affected; an unforeseen accident, however, gave it a finishing stroke. The house in which he lived, which was his own property, was so dilapidated that he was obliged to have some necessary repairs executed. In doing this, unfortunately, he caused the fall of a large piece of wall, and the architect declared it indispensable for the safety of the inhabitants of the house, that a considerable reparation should be made immediately, of which, of course, Joseph would have to bear the expense. This news seemed to strike him like a thunderbolt. He remained all day long motionless, with sunken eyes, paying no attention to anything around him, and in a state of the most profound depression. He was supposed to have been struck with apoplexy, and was bled accordingly. The next night, soon after midnight, his wife saw him light a lamp, and proceed with a quick step towards the closet which contained his 1600 francs. "What are you after?" she said to him. "I just heard some robbers," he replied, "moving my money; let them take care, for I am here." At the same time he opened the closet in a convulsive manner, and stretched his hand out towards the spot where the precious treasure was deposited. Having felt it all round, to satisfy himself that it was intact, he shut the door, locked it, put the key under his pillow, and returned to bed.

Some days after, Joseph brought some workmen to execute the necessary repairs; but, as it frequently happens in similar cases, as they proceeded with the works, additional repairs were found to be needed, so considerable that Joseph who at first flattered himself that but a small portion of his 1600 francs would be required, now began to perceive that the whole, and perhaps something more, would be necessary to defray the expenses. When he saw that all his schemes for becoming rich which he had founded on this secret ready money were being rendered abortive, his reason began to be seriously affected. He repeated continually that he was ruined, that he had to pay the workmen four times more than they deserved. He who formerly had excited them against the rich by dwelling on the smallness of their pay—he wanted to arrest and imprison all his small creditors. As for one man to whom he owed 500 francs, he said he was an aristocrat, without compassion, and deserved the guillotine. He ultimately believed everybody he saw to be either creditors or workmen demanding money, and he fled from them with terror. He imagined all his friends owed him money and would not pay. Sometimes he would pass them with a discontented and fierce air; and when asked the reason of this behaviour he replied, "People who don't pay their debts don't deserve to be spoken to."

He passed few nights without getting up and seeing if his money was safe. "It is his god—his idol," said his wife. "I tremble at the idea of what will happen to him when he is compelled to give it up to pay the workmen." About two o'clock one morning, when his bedroom was but imperfectly lighted by the rays of the moon, his wife saw him take a large knife from one of his pockets, and open it. His eyes were flaming, and his attitude most threatening. The poor woman felt a sudden fear come over her, as her husband jumped out of bed, rushed across the room with a bound, and plunged his knife with a furious thrust into a sack of corn, which had been placed, in the course of the evening, unremarked by him, in the corner of the apartment, and which in his frenzy he had mistaken for a robber. He became subsequently so insane that his friends were obliged to send him to the asylum of Dôle. Some time afterwards I was obliged, as member of the general council, to visit this establishment, and there I found Joseph. He was in a state bordering on dementia, quite indifferent to politics, and always silent. One idea only occupied his mind—he complained always of being ruined and reduced to poverty.

CASE X.—Augustine N., aged thirty-five, of weakly constitution, lymphatic, and nervous temperament; very intelligent. She had received a very good education, and had read a great deal. At the age of twenty-five, while she was reading Lamartine's poem, *Jocelin,* she had an attack of nymphomania, which lasted several months. About this time her parents had refused to allow her to marry a man to whom she was much attached. She completely recovered from her first attack of mental alienation, and regained her former intelligence before the Revolution of February broke out. Towards the end of 1848 her reason became again affected, but the character of her insanity was quite changed. Naturally timid and weak, she became most vividly impressed by the passing political events. She fancied she was constantly threatened with pillage, prison, and the guillotine, and that she beheld cart-loads of dead bodies constantly passing in the street. One Sunday, seeing an altar which had been erected in one of the squares of the city, she ran away, crying out, "Oh! here is the guillotine that has just been put up!" For some months she lived only on bread and water, refusing with horror all kinds of animal food, because she declared the cook had cut her brother into pieces and put them into the *pot au feu.* One of her friends was a fervent legitimist, and spoke to her often of the last scion of the House of Bourbon. Augustine for a long time affirmed herself pregnant with Henry V. One day, when the ideas of death, the guillotine, &c., were more than usually powerful, she was found sitting in the corner of a dark cellar, where she had been for some hours.

At one of my visits, when I was coming close up to her, she pushed me away violently, exclaiming, "You smell of blood—of human flesh; you have just passed near the guillotine;" and she began to smell vigorously at her scent-bottle. After a time she fell into a complete state of dementia, and finished her days in an asylum.

Time will not permit me to describe in detail all the cases of mental disturbance which I observed at the time of the second republic, but I will mention summarily a few of them.

I was myself a witness of a fact which proves to what a height popular exasperation had reached. One evening, about eleven o'clock, I returned home in a carriage and pair, and was received with loud cries of "Down with the rich!" As it was a close carriage I could not be recognised. I was just returning from a poor woman in labour, who was dying from hæmorrhage, and who was only rescued from death through the quickness of the two horses, which a rich neighbour had sent for me, that I might arrive without loss of time.

An old soldier, not very intelligent, harsh, unmannerly, and somewhat brutal, was prejudiced against nobles, priests, and the rich. Pursued by hallucinations, one fine moonlight night he went and broke in with a hatchet the front of one of the principal shops in the city, mistaking it for a castle. One day he wrung the neck of a black cat, denouncing it as a *calotin* (a priest). He always kept a hatchet at his side, to defend himself against the aristocrats.

The wife of a watchmaker, who was a jealous, gloomy, bad-tempered woman, took it into her head that the *Whites* had poisoned her. She felt so great a fire in her interior, that she passed all her time in using clysters of cold water. Even during my visits she never interrupted her operations. If she remained five minutes without a clyster she cried out in a most pathetic voice, "I am burning, I am burning," and seized hold of the syringe. She had always a bucket of cold water standing by her side, from which she could fill it.

A young man of limited intelligence, but very sentimental, when he was in a lonely place one evening with his sweetheart, began all at once to march hastily, and cry out, as if inspired, "I am the Messiah, I will change the face of the earth," &c. I learned this from the lips of the young woman herself, who was so much overcome by the effects of the scene, that her father was obliged to send for me the next morning.

One of my relations had a man-servant who had acquired a great taste for reading. I often met him walking alongside his cart, reading the journal, *La Réforme*. A tenant of mine who slept by chance one night in the same bedroom with this man, told me that he had scarcely been able to close his eyes, because the other would not cease endeavouring to persuade him to become a convert to his ideas of the democratic and social republic. This valet-apostle had, nevertheless, for a master one of the best of men, a true friend of the people; but in his fits of rage he would denounce his employer as an aristocrat, a tyrant, a despot, and a monopolizer.

The sentiment of pride so thoroughly permeated all the masses that every one sought to better his condition: one young woman became so completely imbued with the doctrines of Fourier, that she quite lost her senses. She went about always in man's clothing. One day when she was very much excited, her husband sent for me to come and see her; but it was impossible to do anything for her. She fell into a most violent passion, and declared that until women were permitted to become doctors she would never be attended by one.

From the elector in his blouse, to the representative proud of his seat in the Assembly, it was curious to observe what airs of sovereign majesty each one gave himself. In the midst of so much disturbance there were very few who were not more or less affected by the excitement which seemed present in the atmosphere. There were millions, who, if I may be allowed the expression, were half-mad or a quarter-mad. Few men, in the midst of this noisy turmoil of eccentric opinions and strange theories, were able to raise themselves above it into the region of lucid ideas and healthy conceptions. I will give an example to show to what extent this moral epidemic had extended, even among the best society. One evening, in the assembly at the prefecture of the Jura, groups were formed round two people recently arrived from Paris. Everyone was eager to hear the news that they had brought from the great Babylon, and especially to learn who this Louis Bonaparte was, whose name was in every mouth. One of them said he was only a drunkard who had been often picked-up out of the gutter; another, that he was a kind of small Louis XV., passing his nights in orgies, in the midst of a *Parc aux cerfs* that he had created outside Paris. Nevertheless, these were two men of mature age, worthy citizens, and honourable men, and it was in this way, in those times of deplorable confusion, that they treated the man who was destined to extricate France from her state of chaos, cover her lands with a network of railways, change into fairy residences the majority of her great cities, restore Italy to herself, and, making our soldiers missionaries of civilization, carry the glory of the French name from one end of the universe to the other.

Madness is not only epidemic at certain periods, it is sometimes even contagious, so strong in man is the instinct of imitation. At the end of one of the passages of the Hôtel des Invalides at Paris, an old pensioner was found hung one day; a fortnight after, a second; two months later a third was discovered in the same position. They were obliged to close the passage. At certain epochs also it has been necessary to close the entry to the column in the Place Vendôme, because several persons in quick succession have thrown themselves from the top of the pillar. Who does not remember the History of the

Abderitans ?—of the "possessed" of the middle ages ?—of the convulsionaries of St. Médard ? When I visited the hospital of Bicêtre, in 1839, after the attempts of Fieschi, Alibaud, and others, a great number of insane were admitted, affected with propensities to regicide, or delusions concerning this crime.

The facts related in this memoir lead to some practical conclusions of great importance.

First : Exaggerated ideas of independence, of liberty without bounds, indiscriminate attacks upon great social principles, everything, in a word, that can shake the confidence of the people in their institutions, ought to be most strictly withheld by the government from all publications circulated among the masses. A history of the evils provoked by the subversive ideas of '48 would constitute a *true martyrology of the people*. These ideas are, for the mental state of the population, dangerous miasmata, which develope moral epidemics more dangerous than typhus or cholera. If it is the duty of government to shield the population from the mephitic emanations which engender these diseases, much more ought they to preserve them from the unhealthy theories and corrupting influences which develope such a terrible mental epidemic as that of February, 1848.

Secondly : When a social tempest has caused great disquietude, disturbing even the best balanced minds, the men through whose ability and courage the vessel has been safely brought into port, should be full of indulgence for the ignorant who forgot themselves for a moment in the midst of the revolutionary storm. All the severity of the law should be reserved for those ambitious agitators, who, in the midst of the turmoil, play the part of the ape in the fable, and endeavour to enrich themselves at the expense of the suffering of others.

None but just and healthy ideas should be permitted to circulate among the masses, all moral impurities should be proscribed, as the air destined for respiration should be purified by hygienic rules. Woe to the society which allows all kinds of pernicious doctrines to circulate freely ! for there are always some bad natures which receive them with avidity, even as there are always some temperaments so unfortunately organized that they receive and ripen the germs of every epidemic.

I ought, perhaps, to have published sooner the observations contained in this memoir, but I wished to leave time for the passions raised by the Revolution of February to subside. It is impossible for the historian the day following a great battle, of which he has been a witness, his heart still thrilling with emotion, to sit down and relate the more distressing details of the combat with the calmness and impartiality which his subject demands.

